# Calcific periarthritis of the hand successfully treated with ultrasound-guided barbotage: A case report

**DOI:** 10.1016/j.radcr.2025.04.131

**Published:** 2025-05-29

**Authors:** Andrew Pasion, Hongmin Xu, Caleb Bhatnagar, Alexander Kui, Daniel Heller, Emad Allam

**Affiliations:** Loyola University Medical Center and Loyola University Chicago, 2160 S First Ave, Maywood, IL 60153, USA

**Keywords:** Calcific periarthritis, Calcific tendonitis, Hand, Ultrasound-guided barbotage

## Abstract

Calcific periarthritis is a benign but painful inflammatory process associated with periarticular deposition of calcium hydroxyapatite. It most commonly affects the rotator cuff tendons in the shoulder. Ultrasound-guided barbotage is a safe procedure with a high success rate in treating calcific tendonitis of the shoulder. Calcific periarthritis of the hand is a relatively rare entity. We report a case of a woman with calcific periarthritis in the hand, highlighting its successful management with ultrasound-guided barbotage. This case adds to the few case reports showing the efficacy of barbotage in atypical locations.

## Introduction

Calcific periarthritis is a condition characterized by the deposition of calcium hydroxyapatite crystals within tendons, bursae, capsules, and ligaments, predominantly affecting the rotator cuff tendons in the shoulder. Calcific tendonitis specifically refers to calcium deposits forming within a tendon. Calcific tendinopathy, calcific tendinosis, and calcific tendonitis are often used interchangeably, although tendonitis suggests acute inflammation, tendinosis suggests chronic degeneration, and tendinopathy is a broader term that includes both degeneration and inflammation. Calcific periarthritis typically affects individuals in the age range of 30-60 years, with a higher prevalence among women than men [1]. This condition can lead to significant pain and functional impairment. The clinical presentation may simulate infection, and it has a high rate of misdiagnosis [2].

While calcific periarthritis is relatively common in the shoulder, involvement of the hand and wrist is less common. Calcific periarthritis of the hand and wrist is usually treated with conservative measures such as splints and pain relievers that can lead to pain relief within several days to several weeks [2,3]. In some patients with excessive pain, conservative treatments may not be adequate. Ultrasound-guided barbotage is a treatment option that can be successful in such cases.

Imaging studies, including radiographs and ultrasound, are valuable in accurately diagnosing this condition. Radiographs can reveal the presence of calcific deposits in the region of the tendon or capsule. Ultrasound can give information on the soft or hard nature of the calcification, and has the advantage of real time imaging for interventions [4].

This case report highlights the successful management of a rare presentation of calcific periarthritis of the hand utilizing ultrasound-guided barbotage. The diagnostic evaluation, clinical decision-making, procedural technique, and the subsequent clinical outcomes are discussed. By sharing this case, we hope to provide valuable insights for healthcare professionals involved in the management of this condition.

## Case report

The patient was a 37-year-old woman with no significant past medical history. She presented with right ring finger tenderness and stiffness for 4 days. She was unable to flex or extend her right ring finger due to worsening pain, swelling, and some numbness. Her symptoms had also begun to involve the adjacent right middle finger. She denied any puncture wounds, insect bites, infection, or injuries to her right hand. On physical examination, she had pain and swelling primarily located in the volar aspect of the third webspace between the middle finger and ring finger metacarpophalangeal (MCP) joints. She had self-treated with rest, ice, acetaminophen, and ibuprofen, but without improvement in symptoms.

**Fig. 1:**
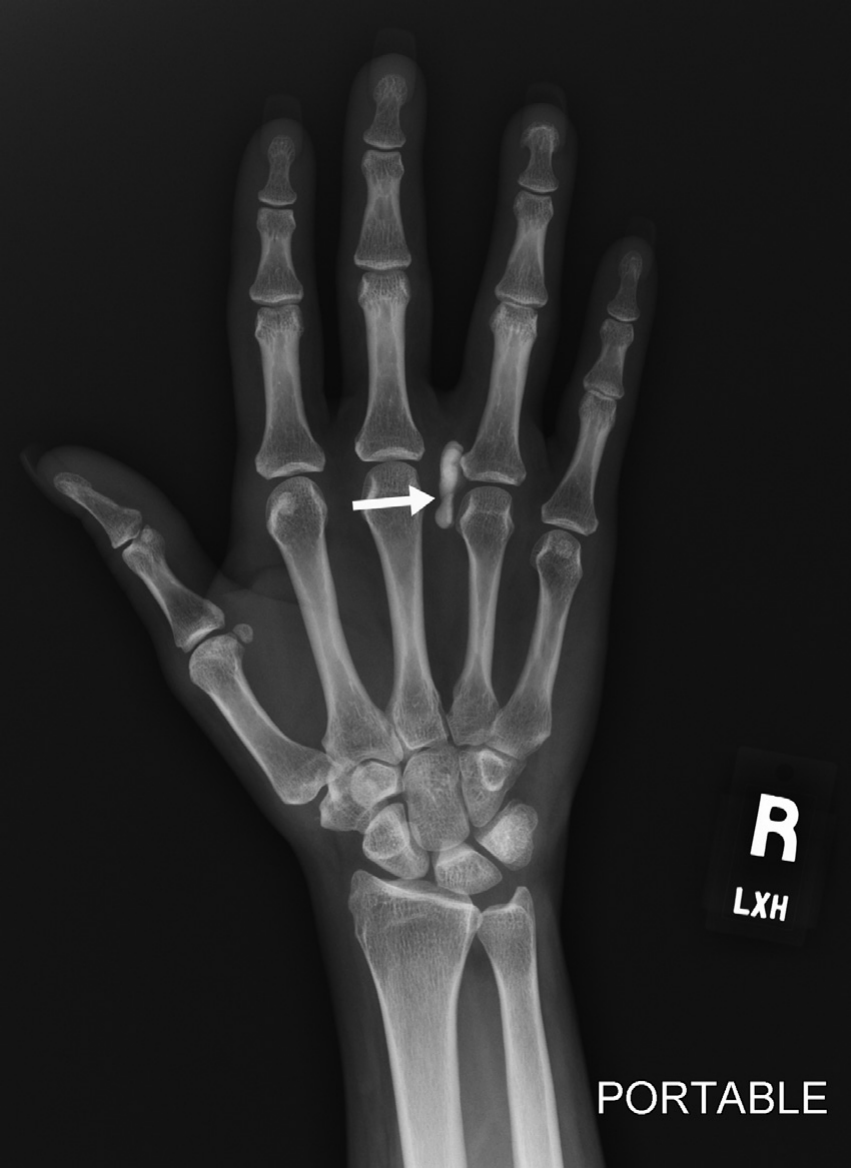
Frontal radiograph of the right hand demonstrates longitudinal calcification in between the third and fourth MCP joint (arrow), with surrounding soft tissue swelling. These findings are compatible with calcific periarthritis.

Radiographs of the right hand revealed a longitudinal hyperdense calcified deposit between the third and fourth MCP joints, with surrounding soft tissue swelling (Fig. 1).

**Fig. 2:**
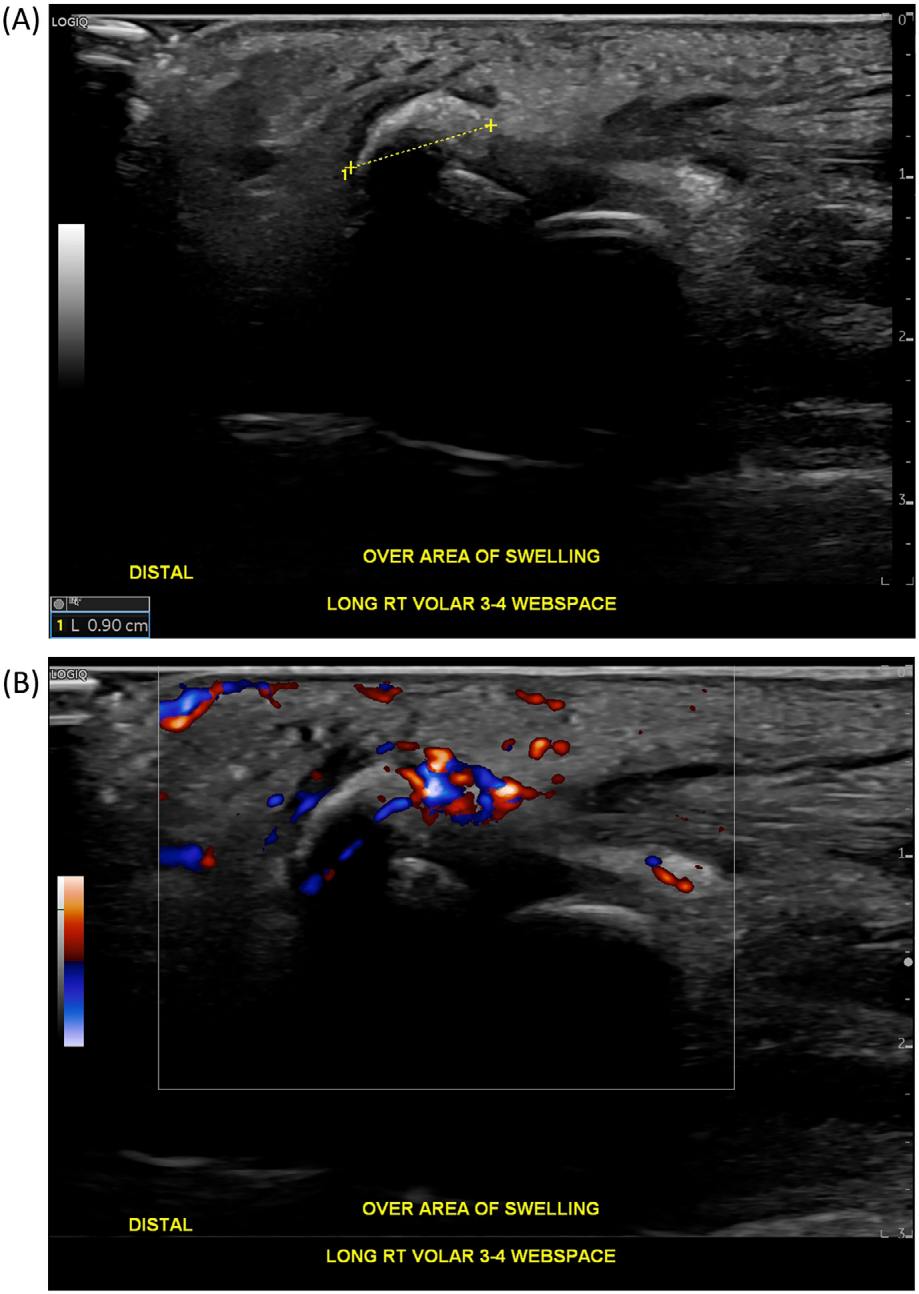
(A) Longitudinal ultrasound image of the right hand demonstrates globular calcification with acoustic shadowing at the third webspace at the level of the MCP joint. (B) Corresponding color Doppler ultrasound image shows hyperemia around the calcification, indicative of inflammation.

Ultrasound examination of the right hand confirmed globular calcification between the middle and ring finger metacarpal heads, specifically at the volar aspect. Additionally, there was intratendinous calcification of the right ring finger flexor tendon, along with surrounding hyperemia indicating inflammation. Altogether, these findings were consistent with calcific periarthritis, more specifically calcific tendonitis with extra-tendinous extension (Fig. 2).

**Fig. 3:**
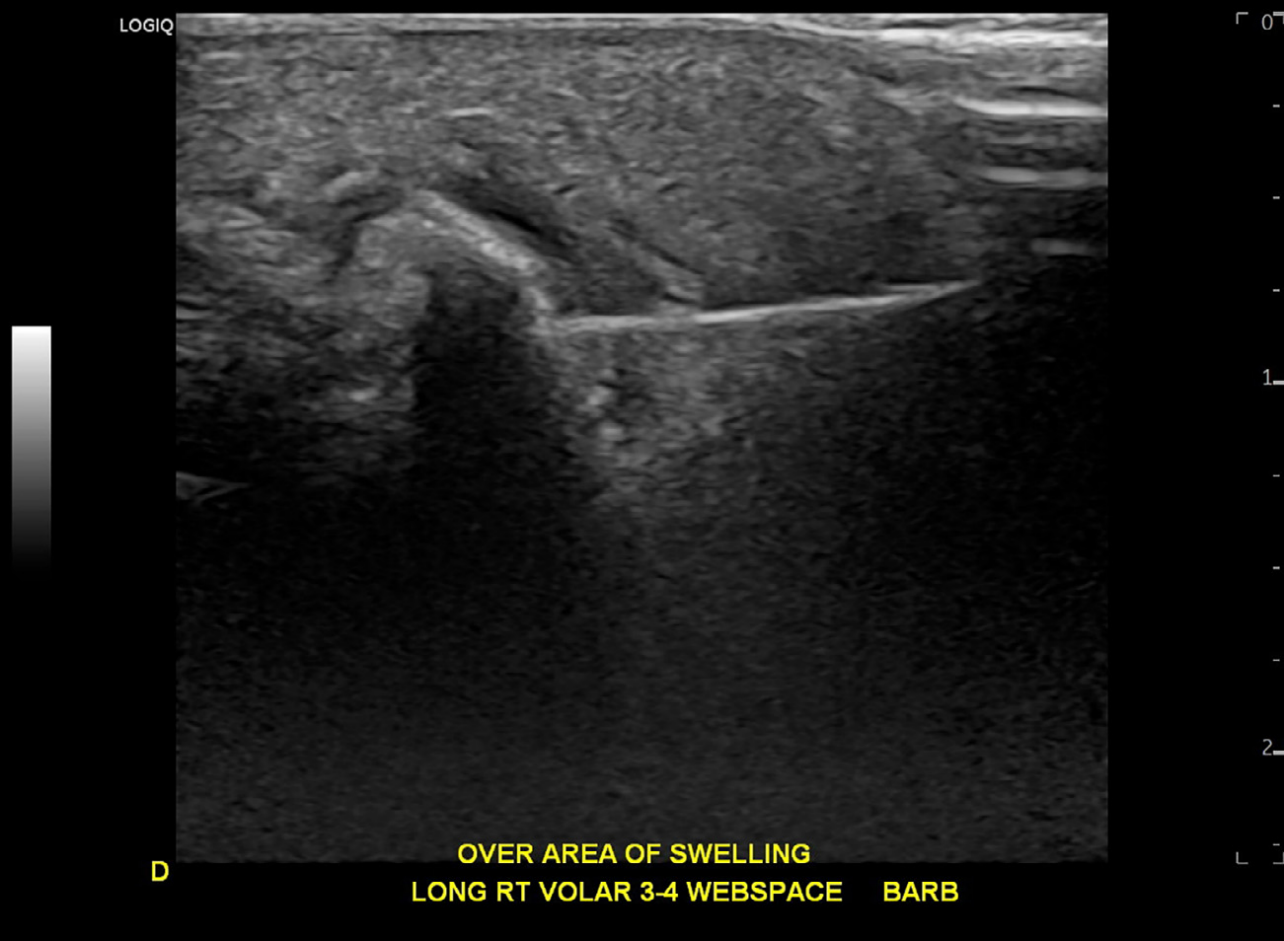
Longitudinal ultrasound image of the right hand demonstrates needle within the calcific focus. Shadowing from the calcium limits visualization of the needle tip on this single image.

Ultrasound-guided barbotage was performed on the same day. Following administration of local anesthesia with 1% lidocaine, a single 18-gauge needle was advanced via the webspace between the middle and ring fingers from distal to proximal into the area of calcification. With normal saline injection and lavage (Fig. 3), the calcification was softened by the end of the procedure, although without visible aspiration of calcium particles into the syringe. The calcification was fragmented via needle fenestration. A mixture of 1 mL of triamcinolone (40 mg/mL), 0.5 mL of 1% lidocaine, and 0.5 mL of 0.5% bupivacaine was subsequently injected in the area of calcification/inflammation under ultrasound guidance. The patient reported approximately 50% relief in pain immediately following the procedure.

**Fig. 4:**
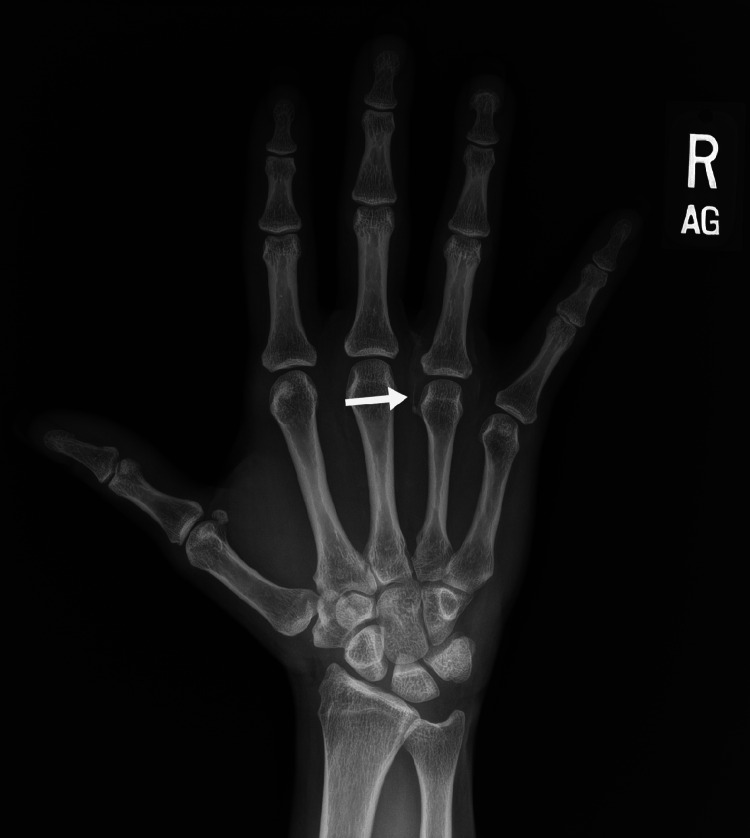
Frontal radiograph of the right hand demonstrates minor residual calcification around the ring finger MCP joint (arrow), markedly improved when compared with Fig. 1.

A follow-up appointment and right hand radiographs were scheduled for a month later. During the follow-up encounter, the patient reported marked improvement in her pain after ultrasound-guided barbotage. On physical examination, surrounding soft tissue swelling had improved. There was only minor tenderness to palpation over the patient’s third webspace, and she was able to make a composite fist. The follow-up radiograph of her right hand revealed near complete resolution of the calcification in the third webspace (Fig. 4) in comparison to the previous radiograph. Mild residual calcifications remained visible around the ring finger MCP joint.

Overall, the patient had significant symptomatic improvement in her pain and decreased calcification in the third webspace after ultrasound-guided barbotage. No further follow-up was considered necessary.

## Discussion

Ultrasound-guided barbotage involves a technique that combines lavage and needling. The lavage component attempts to remove the calcium while the needling component aims to break down the calcium hydroxyapatite deposition in the tendons and soft tissues. To decrease pain and relieve inflammation, this procedure is also combined with the injection of corticosteroids. Barbotage, involving the softening and aspiration/fragmentation of calcifications, has been shown to provide pain relief and promote the resorption of calcium deposits [5]. Most commonly, the procedure is used in treating calcific tendonitis of the shoulder, which affects the rotator cuff tendons [6].

Ultrasound-guided percutaneous irrigation of calcific tendinopathy (US-PICT) has been established as an effective treatment for rotator cuff calcific tendinopathy, offering a minimally invasive alternative to surgical intervention. Chianca et al. provide a comprehensive overview of this technique, emphasizing its role in alleviating pain and restoring function in affected patients [7]. The success of US-PICT in the shoulder suggests its potential utility in managing calcific tendinopathy in other anatomical regions, including the hand and wrist.

Calcific tendonitis/periarthritis of the hand and wrist is rare, with the MCP joint being the most commonly involved joint in the hand, followed by the proximal interphalangeal joint. Conservative therapy with splinting and anti-inflammatory medications is commonly used for this condition [2,8].

Ultrasound-guided barbotage is a simple, quick, and effective way to treat this condition. As a result of barbotage with normal saline, calcium hydroxyapatite reacts with sodium chloride to form sodium hydroxyapatite and calcium chloride, both of which are soluble and thus can be aspirated, leading to immediate pain relief for the patient [4]. To date, less than ten cases of successful ultrasound-guided barbotage for treatment of calcific tendonitis of the wrist/hand have been reported in the literature [4,9,10].

Ahuja et al. demonstrated the efficacy of ultrasound-guided barbotage in treating calcific tendonitis and periarthritis in the wrist and hand through a case series of 6 patients. Four of these patients had flexor carpi ulnaris calcific tendonitis, presenting with tenderness near the pisiform bone, while the remaining 2 exhibited lateral wrist and thumb pain due to thumb MCP and thumb carpometacarpal calcific periarthritis. Radiographs and ultrasound were used to identify calcific deposits, and all patients underwent ultrasound-guided barbotage followed by corticosteroid injections. The treatment resulted in immediate pain relief, with sustained improvement at 6-month follow-up [4]. This prior case series supports the effectiveness of barbotage across different anatomical sites in the hand and wrist. However, our report adds further evidence of its utility in managing periarticular calcifications in the finger MCP region, an area not previously highlighted in their series.

Similarly, Van Demark et al. reported favorable outcomes using lavage and steroid injection for flexor carpi ulnaris calcific tendonitis, emphasizing its role in cases where nonsteroidal anti-inflammatory drugs (NSAIDs) are contraindicated [9]. Ariyaratne et al. further expanded on barbotage as a minimally invasive alternative to surgical intervention in calcific periarthritis causing carpal tunnel syndrome, reinforcing its broader therapeutic potential [10]. In contrast to these cases, our report focuses on the involvement of the MCP region and extra-tendinous extension, highlighting the versatility of ultrasound-guided barbotage for treating periarticular calcifications beyond traditionally recognized sites.

Ultrasound plays a crucial role in detecting tendon calcifications, particularly in atypical locations that may be overlooked on radiographs. While calcific tendonitis most commonly affects the supraspinatus tendon in the shoulder, atypical locations, such as the subscapularis tendon, have been validated using dedicated ultrasound protocols. A study by Antonio et al. emphasized the effectiveness of a structured 5-step ultrasound approach in identifying subscapularis tendon calcifications, which are often missed on conventional imaging [11]. Similarly, in our case, ultrasound not only confirmed the presence of calcific deposits within the third webspace but also identified calcification in the region of the flexor tendon of the ring finger, highlighting the modality's sensitivity in detecting periarticular calcifications around small joints. Integrating standardized ultrasound protocols for the hand, similar to those established for the shoulder, could enhance the early detection and management of calcific periarthritis in uncommon sites.

The successful utilization of barbotage in this case highlights its efficacy as a treatment option for calcific periarthritis. Although no visible calcium particles were aspirated in this case, the procedure resulted in significant pain reduction for the patient. This condition is largely idiopathic, but it is also associated with trauma and systemic and metabolic diseases, such as hypothyroidism, rheumatoid arthritis, diabetes, gout, and pseudogout [2,8]. However, this patient had no known underlying conditions to explain the calcifications. It is important to note that superficial injection of corticosteroids can potentially result in skin hypopigmentation and atrophy. However, this potential side effect was not observed in this case.

While this case report highlights the successful use of ultrasound-guided barbotage for calcific periarthritis in the hand, certain limitations should be acknowledged. As a single case report, the findings may not be generalizable to a broader patient population with varying calcification patterns, symptom severity, or anatomical locations. Additionally, long-term outcomes remain unknown as the patient was not followed up for greater than 1 month. Further studies are needed to assess the durability of symptom relief and the potential for recurrence. Future research should include prospective studies with larger sample sizes to evaluate the efficacy, safety, and long-term outcomes of barbotage for hand calcifications. Comparative studies analyzing barbotage against other treatment modalities, such as corticosteroid injections alone or surgical interventions, would provide valuable insights into the optimal management approach for calcific periarthritis around small joints.

## Conclusion

Calcific periarthritis of the hand is a rare entity. For patients suffering from refractory pain or failing conservative management, ultrasound-guided barbotage is a quick and effective way to treat this condition. Ultrasound not only facilitates diagnosis but also provides real-time guidance for treatment.

## Patient consent

Informed consent for this case was obtained from the patient.
